# Bridging the Species Divide: Transgenic Mice Humanized for Type-I Interferon Response

**DOI:** 10.1371/journal.pone.0084259

**Published:** 2014-01-09

**Authors:** Daniel Harari, Renne Abramovich, Alla Zozulya, Paul Smith, Sandrine Pouly, Mario Köster, Hansjörg Hauser, Gideon Schreiber

**Affiliations:** 1 Department of Biological Chemistry, The Weizmann Institute of Science, Rehovot, Israel; 2 MS Platform, Merck-Serono, (a division of Merck KGaA), Geneva, Switzerland; 3 Helmholtz Centre for Infection Research, Dept. Gene Regulation and Differentiation, Braunschweig, Germany; Friedrich-Alexander-University Erlangen, Germany

## Abstract

We have generated transgenic mice that harbor humanized type I interferon receptors (IFNARs) enabling the study of type I human interferons (Hu-IFN-Is) in mice. These “HyBNAR” (Hybrid IFNAR) mice encode transgenic variants of IFNAR1 and IFNAR2 with the human extracellular domains being fused to transmembrane and cytoplasmic segments of mouse sequence. B16F1 mouse melanoma cells harboring the HyBNAR construct specifically bound Hu-IFN-Is and were rendered sensitive to Hu-IFN-I stimulated anti-proliferation, STAT1 activation and activation of a prototypical IFN-I response gene (MX2). HyBNAR mice were crossed with a transgenic strain expressing the luciferase reporter gene under the control of the IFN-responsive MX2 promoter (MX2-Luciferase). Both the HyBNAR and HyBNAR/MX2-Luciferase mice were responsive to all Hu-IFN-Is tested, inclusive of IFNα2A, IFNβ, and a human superagonist termed YNSα8. The mice displayed dose-dependent pharmacodynamic responses to Hu-IFN-I injection, as assessed by measuring the expression of IFN-responsive genes. Our studies also demonstrated a weak activation of endogenous mouse interferon response, especially after high dose administration of Hu-IFNs. In sharp contrast to data published for humans, our pharmacodynamic readouts demonstrate a very short-lived IFN-I response in mice, which is not enhanced by sub-cutaneous (SC) injections in comparison to other administration routes. With algometric differences between humans and mice taken into account, the HyBNAR mice provides a convenient non-primate pre-clinical model to advance the study of human IFN-Is.

## Introduction

Type I Interferons (IFN-Is) comprise a family of sixteen human cytokines, which collectively feed into a signaling network through binding to two-receptor components, IFNAR1 and IFNAR2. Formation of the ternary complex induces a cascade of down-stream activities through the reciprocal phosphorylation of Tyk2 and Jak1 that are associated to the receptor. Subsequently, they phosphorylate several tyrosine residues in the membrane-distal intracellular domains of IFNAR1 and IFNAR2, which, in turn, recruit and activate STATs through their phosphorylation (reviewed by [1]). Only a few hundred IFNAR receptors are typically expressed per cell [2], [3], their number impacting upon the cell's ability to respond to IFN-I signaling. Perhaps for this reason, IFN-I signaling is particularly sensitive to perturbation by negative feedback loops, dampening the response with time [4]–[6], [2], [7]–[10].

Receptor binding activates a diverse set of overlapping biological outputs such as anti-viral, anti-cancer, and a wide range of innate and adaptive immunity [11]–[13]. How such diverse outputs can be induced through a single heterodimeric receptor-signaling complex is still an open question. However, it has been shown that differences in receptor-binding affinities of the different IFN-I cytokines, their concentration and duration of activation are important factors. Twelve of the sixteen IFN-Is belong to the IFNα subtype. These, together with all but one of the remaining human homologs IFN**ε**, IFNκ, IFNω all bind the IFNAR1 receptor with low affinity, and when complexed together with IFNAR2 induce the activation of a cascade of anti-viral and pro-immunoregulatory inflammatory effects. Conversely, IFNβ the single high-affinity IFN-I in humans, can exert additional physiological functions such as the induction of growth-arrest and apoptosis at low concentrations [3], [14], as well as induction of anti-inflammatory activity [15].

The high affinity receptor binding IFNβ induces an anti-proliferative response with a 20–200 decreased EC_50_ in comparison to IFNα [3], [16]. Increased receptor-binding affinity is the major determinant differentiating the biological activity of IFNβ from that of the remaining IFN-Is. This is supported by the finding that a variant of human IFNα2 genetically engineered to bind tightly to IFNAR1 converts this “IFNα” into a superagonist with anti-proliferative potency surpassing that of IFNβ [17], [18]. This tightly-binding superagonist was generated by exchanging three amino acids receptor-binding interface (HEQ→YNS). To IFN-YNS, we next added the five amino acid carboxyl-tail sequence borrowed from the cytokine IFNα8, which additionally strengthens the binding to the IFNAR2 receptor by 15-fold [3]. Our variant YNS and YNSα8 mutants bind the human IFNARs with higher affinity than any known IFN-I found in nature, and to our knowledge better than any other IFN-I engineered mutant. Their potency in inducing anti-proliferative activity relates directly to their increase in binding affinity. These data all provide a mechanistic explanation to how high affinity IFNβ differs phenotypically to the remaining low affinity members of the IFN-I cytokine family, but what biological properties phenotypically separate the different low affinity IFN-I members still remains a question largely unresolved.

Ultimately, the activity of a particular IFN-I will relate to its amino acid sequence governing its three dimensional space, particularly so at the receptor binding interface for IFNAR1 and IFNAR2. The recently determined ternary structure of human IFN-IFNAR bound complex has revealed a surprising similarity in the interface of cytokine/receptor binding for both high and low affinity cytokines [19], again suggesting that binding affinity rather than structural differences are responsible for differential activation. Although IFN-I signaling is evolutionarily conserved amongst vertebrates [20], genes within this family are subject to strong genetic divergence. The protein sequences of both IFNAR1 and IFNAR2 for humans and mice share only 50–51% sequence identity respectively, with the divergence extending to the regions of cytokine binding. Thus, although sufficient data has emerged to demonstrate that IFN-I signaling in both humans and mice results in similar activation of phenotypic outputs, the fine-tuning dynamics of how the divergent cross-species receptors are being activated by their respective cytokines remains a matter to be resolved.

At a practical level, the cross-species barrier separating humans and mice results in the inability to correctly study human IFN-Is in non-primates. Resultantly, even though there are hundreds of clinical trials taking place today using Hu-IFN-Is or long-life variants of them, these trials have been formulated without the advantage of first testing them directly in the often powerful pre-clinical rodent setting. To overcome this, we generated a transgenic mouse that harbors humanized type I interferon receptors, allowing the cross-species study of human type I interferons (Hu-IFN-Is) in mice. These “HyBNAR” (Hybrid IFNAR) mice encode transgenic interferon receptors, whose extracellular and cytoplasmic segments are encoded by human and mouse sequences respectively. This allows the binding of human IFN-Is to the recombinant receptors but once activated, transduces their signal effectively in a mouse cellular environment. In this paper, we describe the generation of the HyBNAR mice and a mouse cell line harboring the HyBNAR transgenes, and demonstrate to proof-of-concept their ability to induce a sensitive response to Hu-IFN-Is, and provide some insight into suggested dosing regiments, this which differs significantly to the human scenario.

## Experimental Procedures

### Mouse Stocks, Maintenance and Ethics Statement

Stocks of C57BL/6 mice were purchased from Harlan Laboratories, Israel. Mice were maintained on site in the Weizmann Institute of Science animal facilities. All mouse experiments were performed strictly according to the Weizmann Institute of Science ethics committee guidelines and permissions (IACUC permit numbers 02160412-3 and 012150412-3).

### HyBNAR construct design

**Figure 1:**
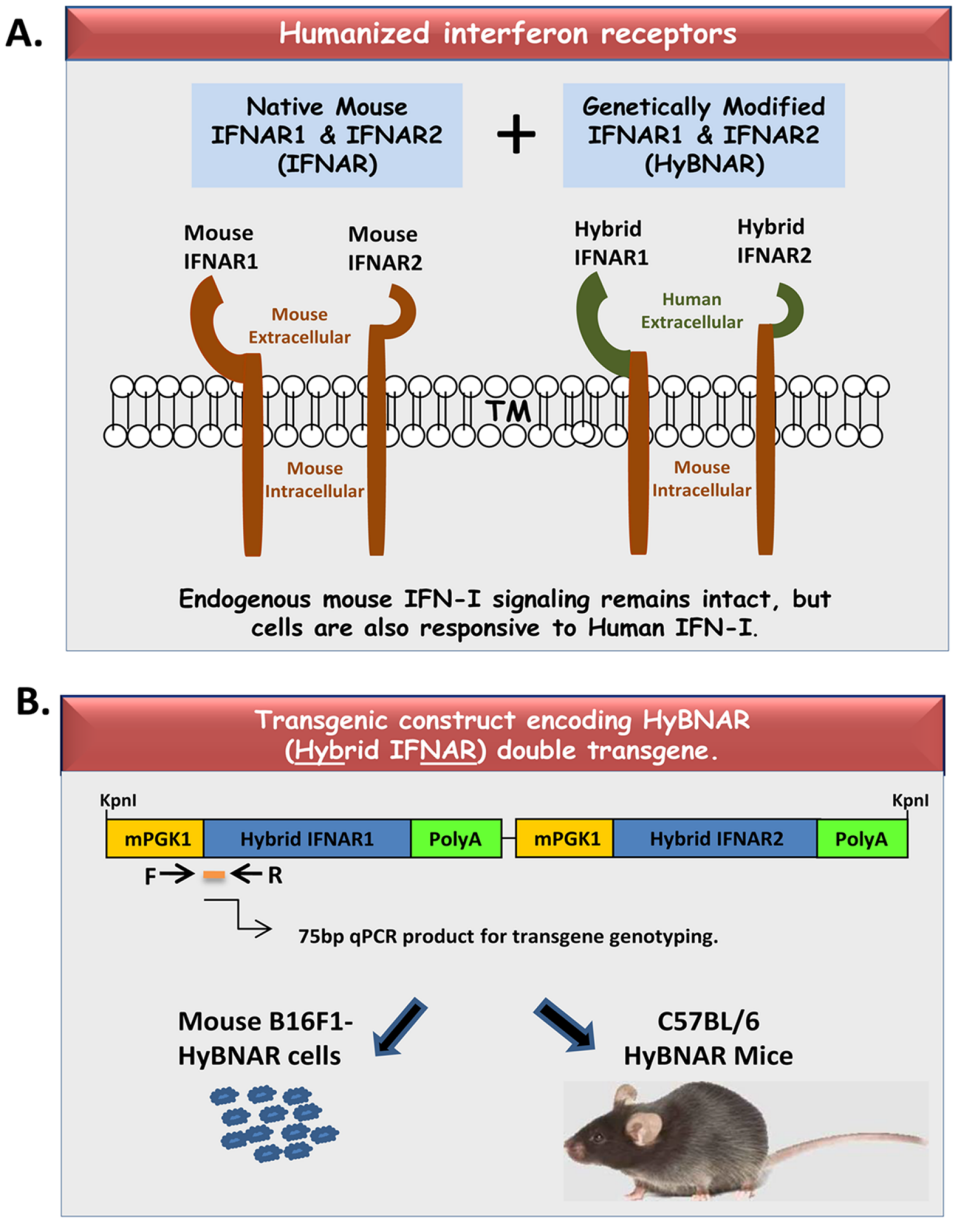
Transgenic HyBNAR Mice and Cell Line. IFN-Is transduce their signals to recipient cells via their binding to and transactivation of a heterodimeric cytoplasmic receptor complex containing IFNAR1 and IFNAR2. But human IFN-Is trans-activate the mouse IFNARs poorly. (A) To overcome this, we have introduced chimeric IFNAR receptors encoding the extracellular domains of IFNAR1 and IFNAR2, fused to their respective transmembrane and intracellular components of mouse origin. By using a regular transgeneisis methodology (IE: not by targeted IFNAR knock-in) we can ensure that regular mouse IFN-I signaling is not disrupted. (B) Diagram of the HyBNAR (Hybrid IFNAR) transgenic construct. Hybrid human and mouse IFNAR1 and IFNAR2 sequences were independently placed under the control of the weak but ubiquitously expressed mouse PGK1 promoter. The two transgenes were then linked together into a single construct. By stable transfection of this construct into the B16F1 mouse melanoma cell line or by pro-nuclear injection into the fertilized embryos of C57BL/6 donors, HyBNAR expressing cells and transgenic mice were generated.

A RF-cloning strategy was employed to PCR the transmembrane and intracellular domains of mouse IFNAR1 (a gift from M. Rubinstein, the Weizmann Institute) to be inserted in-frame into the corresponding human IFNAR1 cDNA sequence (accession #BC021825, cloned into pCMV-Sport6; Open Biosystems) using the primers AGTGACGCTGTATGTGAGAAAACAAAACCAG-GTCAGAATCTTTTATTGTC & CCTGCTGAAAAACCTTATACTTGACACAGTTCATTTCTGG-TCAGCAGAGAAGAGCTGGCTCTGTC). A Similar strategy was used to generate chimeric IFNAR2 but in this case replacing the human 5′ IFNAR2 sequence into a plasmid encoding the full mouse gene (Open Biosystems, Accession #BC071225) primers AGCAAAACGGACTTAAGAGCTGAGCAGGATG-CTTTTGAGCCAGAATGCCTTC and CGAAGTAGTTATTCCTACTATAGCAGATTCTGATAATCC-TGATTCCTGGCCAGGTGGAAGGA. Both chimeric transgenes were independently fused to a 512 bp sequence encoding the mouse PGK1 promoter using primers ATTCTACCGGGTAGGGGAGGCGC & AGGTCGAAAGGCCCGGAGATGAG. The mouse Growth Hormone polyadenylation sequence was then inserted downstream to the termination stop codon of both these transgenes. The two constructs were placed in tandem, generating a double-transgene 7.6 Kb in length (Fig. 1) and were sequence-verified before use.

### Cell lines and transgenic mice

The HyBNAR construct was stably co-transfected into mouse B16-F1 melanoma cells (ATCC) along with a carrier plasmid (pEGFP-N1) and subjected to puromycin selection after which resistant clones were further selected by live FACS sorting for human IFNAR2. Transgenic HyBNAR#1 & HyBNAR#2 mouse strains were generated through the ES-cell transfection approach and generation of lines through chimeric mouse intermediates (Regeneron Pharmaceuticals). The remaining HyBNAR mouse strains (inclusive of HyBNAR#11 chosen for further studies) were generated by pronuclear microinjection of KpnI linearized transgene into purebred C57BL/6 embryos and maintained in this purebred background using breeders supplied by Harlan Laboratories (Israel). Transgenic HyBNAR mice were genotyped by TaqMan-based genomic PCR using the external probes CGGGAATTCAGGTCGAAAGG & CATTCTGCACGCTTCAAAAGC and the internal probe 5′-/56-FAM/GCG CTG TTC/ZEN/TCC TCT TCC TCA TCT C/3IABkFQ/-30′ (IDT). VIK-labeled mouse TERT genomic DNA (Applied Biosystems) was used as a reference gene in order to determine copy-number. MX2-LUC C57BL/6 mice (Pulverer et al., 2010) were kindly provided by Mario Köster (Helmholtz-Zentrum für Infektionsforschung GmbH). Mice were maintained in the Weizmann Institute animal Facilities.

### Reagents

Monoclonal anti-human IFNAR1-EC AA3 antibody was a gift from Biogene. Monoclonal anti-human IFNAR2-EC 117.7 antibody was a gift from Daniela Novick. Both of these antibodies do not cross-react with mouse IFNARs and hence specifically stain the transgenic HyBNARs. Antibodies for Western Blot detection of total-STAT1 as well as Phospho-STAT1 (Tyr 701) were purchased from Santa Cruz (sc-C111 & sc-7988-R respectively). Human IFNα2, IFN-YNS and YNSα8 were expressed and purified as described (3). Mu-IFNβ (recombinant-bacterial) and mammalian-cell-derived human IFNβ were both provided by Merck-Serono, Geneva. D-Luciferin was purchased from Regis Technologies, USA). Tri-Reagent was purchased from MRC-Inc.

### Competitive binding assay

IFN-YNS was labeled with 125I by using the chloramine T iodination method as has been described (3). For competition studies, the indicated cold ligands were incubated with a 100-fold molar excess together with the radiolabelled IFN-YNS.

### B16F1 cell Bioassays

For the Anti-proliferation study, B16-F1 cell clones were seeded at 5000 cells/well into 96-well plates and grown overnight before addition of indicated concentrations of IFN-Is. The cells were incubated for a further 72 hours before being stained with 5 mg/ml Crystal violet (Sigma) in 70% Ethanol. After staining the plates were rinsed in water and air-dried before adding resuspension buffer (0.1 M NaCitrate, 50% EtOH, pH 4.2) for reading at ODs at 540 nM. Both FACS labeling to quantify surface expression of transgenic IFNAR1 and IFNAR2, and phospho-STAT1 western Blot analysis were performed as described (3).

### Quantitative PCR (qPCR) analyses

RNA was extracted from liver and B16F1 cell clones by Tri-Reagent protocol (MRC Inc.). Random-oligonucleotide directed cDNA synthesis was generated from 1 ug RNA per 20 ul reaction (High Capacity cDNA Reverse Transcription kit; Applied Biosystems). Forward and reverse oligonucleotide probes for mouse genes used in this study include MX1; (GGATAATCAGAGGGATCTGTCTCC & AGGCATTAATAAACCCTGCTACCT) Trail (TCACCAACGAGATGAAGCAG & TGGAGTCCCAGAAATCCTCA) and the reference gene HPRT1 (AGCAGTACAGCCCCAAAATG & GGCCTGTATCCAACACTTCG). DNA was amplified using Power SYBR Green and analyzed using a 96-well 7300 Real-Time PCR System (Applied Biosystems).

### Luminescence Studies

For live studies, HyBNAR/MX2-LUC and MX2-LUC mice were injected IP with 100 ul D-Luciferin solution (60 ug/ml, in PBS) 10 minutes prior to luminescence measurements. Mice were lightly anaesthetized with isoflurane and images taken using the IVIS Spectrum optical imaging device (Caliper Life Sciences Inc.) with devoted quantification software (Living Image 4.1) provided by the same supplier. For tissue analyses, IFN-treated mice were perfused with PBS, and tissues were extracted and stored at −80°C before being weighed and homogenized in 10× volume/weight using Reporter Lysis Buffer (RLB); Promega (Cat.# E3971). Immediately prior to luciferase readings, cell extracts were diluted a further 10-fold in RLB and 10 ul samples were placed in 96-well plates. Immediately prior to readings, 50 ul of luciferin buffer (20 mM Tricine, 0.1 mM EDTA, 1.07 mM (MgCO_3_)_4_Mg(OH)_2_*5H_2_O, 2.67 mM MgSO_4_, 3.3 mM DTT, 270 µM Coenzyme A, 470 µM luciferin and 530 µM ATP, pH 7.8.) was injected into each well and luminescence was measured using a Modulus Microplate Luminometer (Turner Biosystems).

For luciferase quantification of live animals, Luciferin-treated mice were photographed with IVIS spectrum as described above. For quantification of the live image, a circular region of interest (ROI) of predefined size was chosen and centered upon highest intensity detected (IE: the liver region) for each mouse. Average radiance was then calculated using Living Image version 4.1 software by Caliper Life Sciences.

## Results

### HyBNAR-transfected B16F1 mouse melanoma cells are humanized for IFN-I signaling

**Figure 2:**
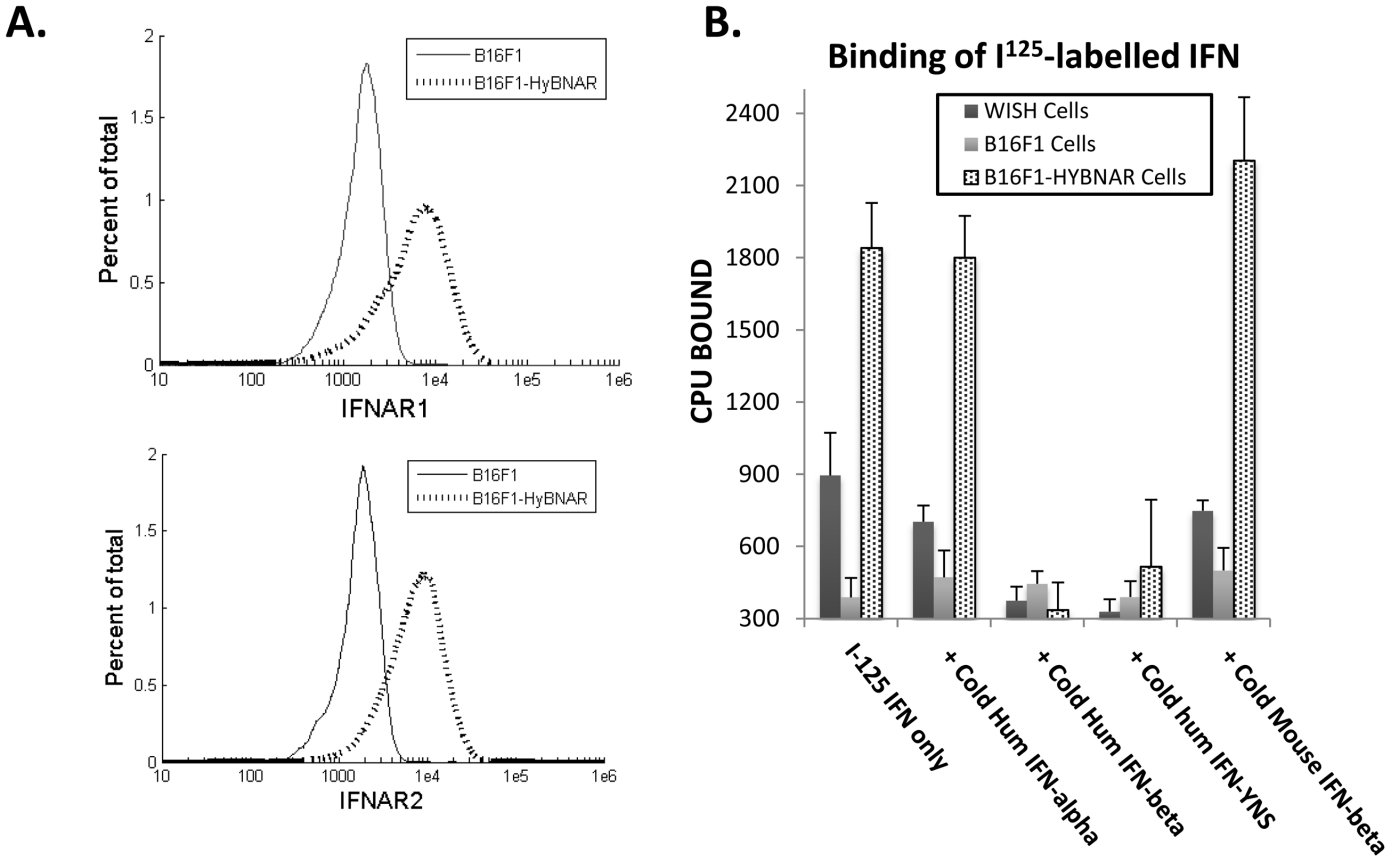
Transgene expression on B16F1-HyBNAR mouse melanoma cells and its binding to human IFN-I. (A) FACS analysis of B16F1-HyBNAR cells using antibodies that specifically bind to the human variants of the extracellular domains of IFNAR1 and IFNAR2. Specificity of binding is demonstrated by the staining of non-transfected mouse B16F1 control cells. (B) YNS, an engineered tight-binding variant of IFNα2 was radiolabelled and incubated with mouse B16F1, B16F1-HyBNAR and human WISH cells. Tightly binding radiolabelled YNS could not be displaced by co-incubation with 100× molar excess of lower affinity Hu-IFNα2a, although high affinity human IFNβ and YNS completely displaced the hot ligand. Importantly, 100× molar excess of mouse IFNβ did not displace binding of hot YNS in this system.

The HyBNAR construct constitutes the human-encoded extracellular domains of both IFNAR1 and IFNAR2, whereas their transmembrane and intracellular domains are encoded by mouse sequence (Fig. 1A). Both transgenes were individually placed under the control of the mouse PGK1 promoter in order to drive constitutive low level expression of the humanized IFNAR1 and IFNAR2 genes (Fig. 1B). The HyBNAR double-transgene was first stably transfected into the murine melanoma B16F1 cell line to test and validate our construct design. Cell surface expression of the HyBNAR receptors for B16F1-HyBNAR cells was detected by live FACS staining using antibodies directed against the extracellular domains of human IFNAR1 or IFNAR2 (Fig. 2A). Comparative staining of human WISH and MDA-MB231 cells (data not shown) suggest that the HyBNAR expression levels are in the same range as IFNAR1 and IFNAR2 expression in human cells. To test for the ability of the B16F1-HyBNAR cells to competitively interact with human IFN-Is, binding of the high affinity IFNα2-YNS mutant to the HyBNAR receptors was evaluated. ^125^I-YNS bound specifically to B16F1-HyBNAR cells as demonstrated by increased levels of radioactive signal detected in comparison to background levels measured for non-transfected B16F1 cells (Fig. 2B). The strong binding of YNS to human IFNAR1 was displaced by competition binding with 100-fold molar excess of non-labeled YNS itself, or high affinity IFNβ, but not by weaker binding IFNα2A (Fig. 2B). Radiolabelled ^125^I-YNS could not be displaced by 100-fold excess of mouse IFNβ (Mu-IFNβ), further supporting the species barrier between human and mouse IFN-I binding (Fig. 2B).

**Figure 3:**
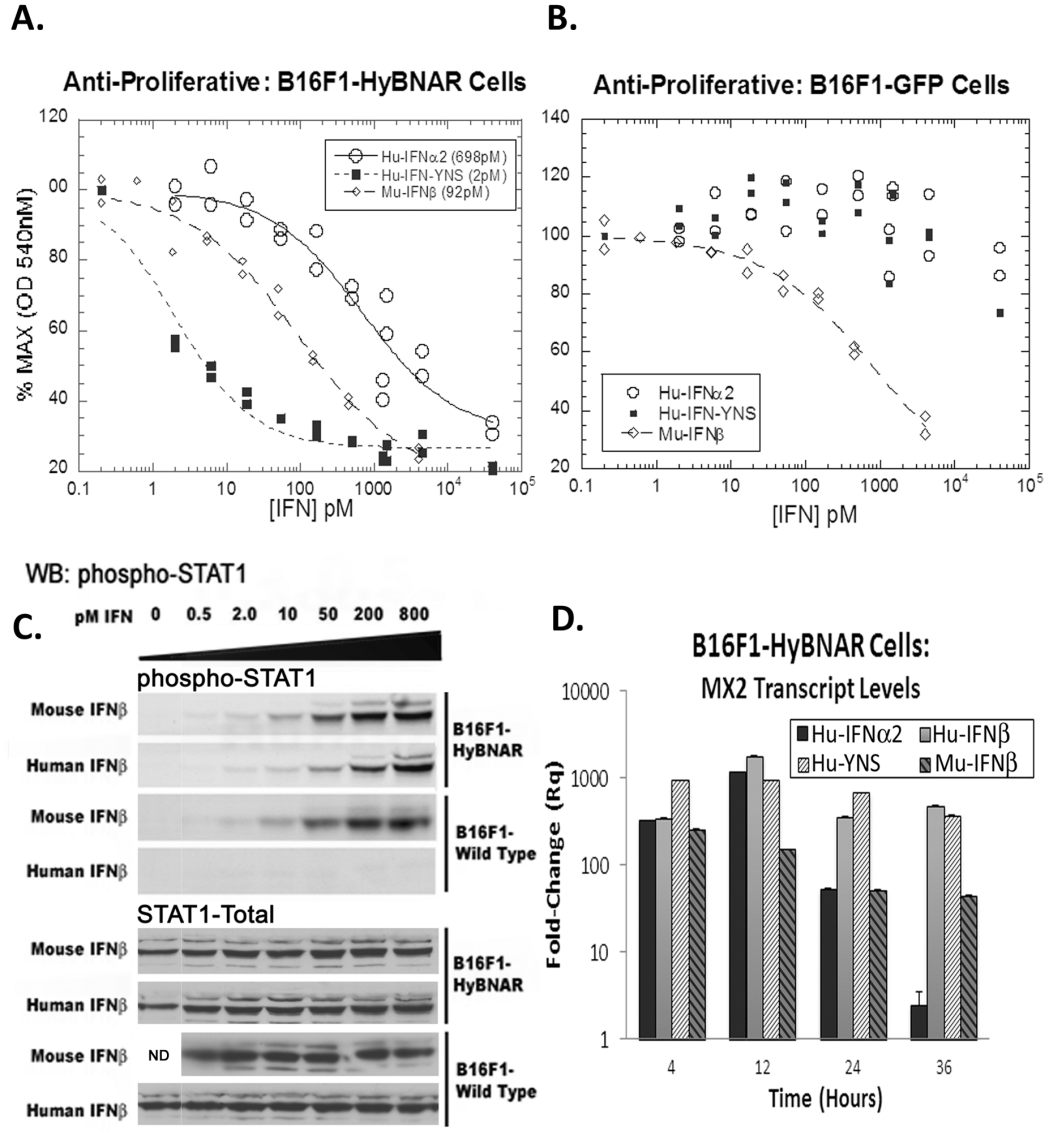
B16F1-HyBNAR mouse melanoma cells are sensitive to human IFN-Is. (A & B) Mouse B16F1 melanoma cells were stably transfected with either the HyBNAR double transgenic construct, or a control GFP vector and tested for their anti-proliferative dose response to Hu-IFNα2, Hu-IFN-YNS and for comparison, mouse IFNβ. (A) HyBNAR transfected B16F1 cells were responsive to both human and mouse IFN-Is (EC50 values shown in parentheses) (B) In the GFP-transfected B16F1 control cells however, the human ligands only promoted a minor loss in proliferation and when administered at very high concentrations. (C) B16F1-HyBNAR and non-transfected control cells were incubated with an increasing dose of both human and mouse IFNβ. After 45 minutes incubation the cells were lysed and analyzed by Western Blot detection for phosphorylated STAT1. Total STAT1 measurements are shown as control. (D). B16F1-HyBNAR cells were treated for the indicated time-points with 100 pM human and mouse IFN-Is. Measurements of the IFN-response gene MX2 were performed by qPCR analysis. Relative fold-change was determined in comparison to untreated cells and normalized using the reference gene HPRT1. Error bars represent standard error of duplicate measurements.

To evaluate the biological activity of the B16F1-HyBNAR cells against Hu-IFNs in comparison to B16F1 cells harboring GFP, an anti-proliferative dose response to Hu-IFNα, high affinity Hu-IFN-YNS, and Mu-IFNβ as positive control was conducted. The B16F1-HyBNAR cells demonstrated high sensitivity to the superagonist ligand IFN-YNS (EC50 2 pM), a value similar to that measured for human cancer cell lines [3]. Hu-IFNα2 also drove antiproliferative activity in the HyBNAR expressing mouse cells, but with a decreased potency of two orders of magnitude (Fig. 3A), which is in line with findings for human cancer cells. In contrast the B16F1-GFP control clone was only sensitive to Mu-IFNβ anti-proliferative response, with minimal effects noted for the human IFN-Is when administered at extremely high doses (Fig. 3B). Thus we demonstrate here that expression of the HyBNAR construct converts this mouse melanoma cell line to being sensitive to Hu-IFN-I.

A major component of IFN-I signaling is via the recruitment and activation of STAT transcription factors [21]. B16F1 and B16F1-HyBNAR cells were treated with increasing doses of either human or mouse IFNβ for 45 minutes and assayed for STAT1 activation by western-blot detection for the phosphorylated STAT1 isoform. Both human and mouse IFNβ activated STAT1 in the B16F1-HyBNAR cells to a similar degree and in a dose-dependent manner (Fig. 3C). However the non-transfected parental B16F1 cells were only responsive to the mouse IFNβ. We did not see cross-activation of STAT1 after Hu-IFN-I stimulation in the parental B16F1 mouse cells, indicating that at least for the maximum concentration of IFN used in this assay (800 pM) the endogenous IFNARs are not being activated by the human cytokine. These data thus support that the activation of STAT1 by human IFNβ is through the HyBNAR receptors, as no activation of parental B16F1 cells was detected when using the human cytokine under these conditions (Fig. 3C).

IFN-I induces the activation of more than a thousand genes [22], [23]. The anti-viral genes MX1 and MX2 are particularly well described experimental markers, which are strongly up-regulated after IFN-I exposure [24], [25]. B16F1-HyBNAR cells were treated for 4, 12, 24 or 36 hours with 100 pM of either human IFNα2, IFNβ, YNS or Mu-IFNβ and transcript levels of the MX2 gene were measured by quantitative PCR (qPCR). Both mouse and human IFN-Is robustly activated MX2 in the B16F1-HyNBAR cells (Fig. 3D). After 36 hours of incubation we noticed a down-regulation of IFNα2-induced signal, as also reported previously [17], [9]. Conversely, MX2 mRNA was maintained at high level over the time-course of this assay when incubated with high affinity Hu-IFNs, a finding consistent with study of human cells lines. In the parental B16F1 cells, Hu-IFNs did not elicit an activation of MX2 expression (data not shown), again supporting the preferential activation of human IFN-Is via the recombinant HyBNARs.

### HyBNAR Mice Respond to human IFN-I

**Figure 4:**
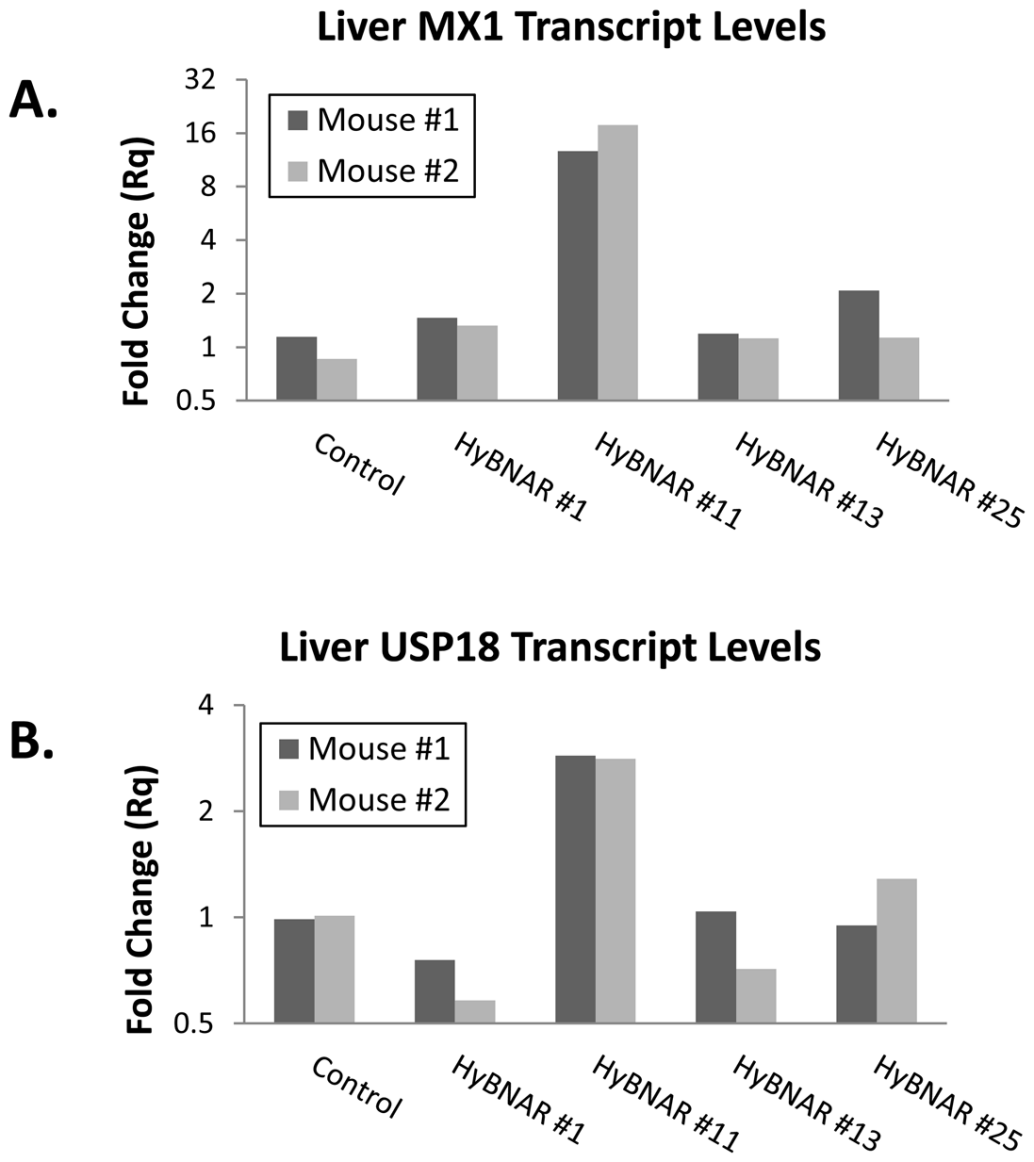
Response of different HyBNAR mouse strains to human IFNβ stimulation. Two representative mice from four independent transgenic HyBNAR strains were injected IV. with 400**β**. Six hours later, livers were collected and RNA was extracted for qPCR measurements. (A) Transcript levels for the IFN-response gene MX1 and (B) USP18. Relative fold-change was determined in comparison to untreated cells and normalized using the reference gene HPRT1.

C57BL/6J mice were used for the generation of transgenic HyBNAR strains using either ES cell transfection (HyBNAR strains #1 & #2) or pro-nuclear microinjection (strains HyBNAR #11, #13, and #25). We did not choose to apply a gene knock-in approach as the natural mouse IFNARs are important to maintain endogenous mouse IFN-I signaling (autocrine and paracrine). TaqMan-based genomic qPCR was performed to determine gene copy number for the different strains of HyBNAR mice, which were found to range from approximately one to sixty five copies of the HyBNAR double transgene (Fig. S1). Initial screening for activity of the HyBNAR transgene was performed in transgenic progeny of the different HyBNAR strains by measuring the activation of the IFN-I response gene MX1 from liver extracts taken six hours after injection of Hu-IFNβ. HyBNAR strain #11 which harbors the lowest transgene copy number (Fig. S1), demonstrated sustained activation of IFN-I response markers such as the genes MX1 and USP18 (Fig. 4). The two HyBNAR mouse strains generated by ES cell electroporation route (HyBNAR#1 and HyBNAR#2) initially exhibited responsiveness to Hu-IFNs, but lost their IFN-I responsiveness after 2–3 generations of backcrossing from their transgenic founders (data not shown) and thus were discontinued. All further studies were conducted with the C57BL/6-HyBNAR#11 strain, which we refer to henceforth as the “HyBNAR mouse”.

### Tracking IFN-I activity in HyBNAR Mice

**Figure 5:**
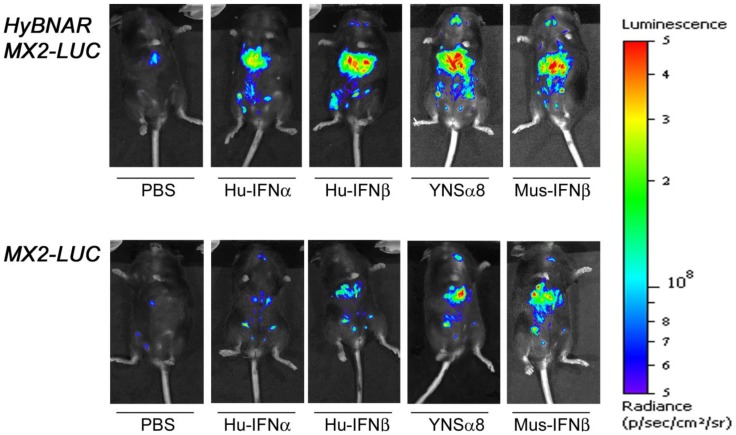
Luciferase signal in HyBNAR/MX2-LUC and MX2-LUC mice in response to different IFN-Is. Mice were injected IP with 1.0-Is. At T = 3.0 hours live animals were injected with luciferin, anaesthetized and live luminosity was measured by an image capturing (IVIS spectrum) device. For comparative purposes MX2-LUC mice without the HyBNAR transgene were also analyzed (bottom panel).

**Figure 6:**
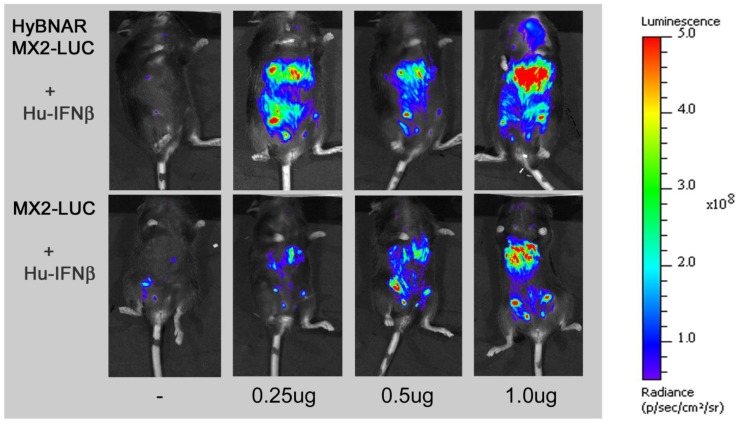
HyBNAR/MX2-LUC mice are both sensitive and responsive to human IFN-Is. Transgenic mice expressing the luciferase reporter gene under the control of the MX2 promoter (MX2-LUC) were interbred with the HyBNAR mice. The HyBNAR/MX2-LUC mice (upper panel) were injected IP with increasing concentrations of human IFN**β**. After 6 hours, mice were injected with luciferin, anaesthetized and live luminosity was measured by an image capturing device (IVIS spectrum). For comparative purposes MX2-LUC mice without the HyBNAR transgene were also analyzed (bottom panel).

A transgenic mouse line that expresses the luciferase reporter gene under the control of the IFN-I responsive MX2 promoter was previously reported, allowing for rapid assessment of the *in vivo* pharmacodynamic effects of Mu-IFN-I injections [26]. These mice, named MX2-LUC, were obtained and crossed with the HyBNAR mice. Double heterozygous transgenic HyBNAR/MX2-LUC mice were generated and adult mice were injected with 1.0 ug of Hu-IFNα, Hu-IFNβ, Hu-YNSα8, or Mu-IFNβ and analyzed by live imaging for bioluminescence. For control measurements, MX2-LUC mice not harboring the HyBNAR transgenes were similarly treated (Fig. 5). Presence of the HyBNAR transgene rendered the mice sensitive to activation of the MX2 promoter-driven luciferase, with the highest levels of signal emanating from what is likely to be the liver. However, we detected some basal activation of MX2-LUC by Hu-IFN-Is also for mice that are not transgenic for HyBNAR (Fig. 5). This contrasts to our findings for the B16F1 cell lines in which no basal activation of wild type cells by Hu-IFNs was detected unless when administered at extremely high concentrations (Fig. 3B). To evaluate the difference in responsiveness towards Hu-IFN-Is in HyBNAR versus wild-type mice, we next injected progressively lower doses (1.0, 0.5 and 0.25 ug) of Hu-IFNβ. Injection of this Hu-IFN-I generated very strong up-regulation of the luciferase signal in a dose-responsive manner for the HyBNAR/MX2-LUC mice (Fig. 6). Injections of the same concentrations of Hu-IFNβ into MX2-LUC mice controls showed also a gradual progressive activation of luciferase signal, however, much lower than obtained in the HyBNAR mice (Fig. 6). We rule out the possibility that contaminants within our Hu-IFN preparations are the cause of the low level of IFN-I activation observed in the non-HyBNAR mice (e.g. residual LPS can induce activation of IFN-I expression in TLR-expressing cells) as the recombinant Hu-IFNβ was derived from mammalian cells (Merck-Serono; human injection quality).

**Figure 7:**
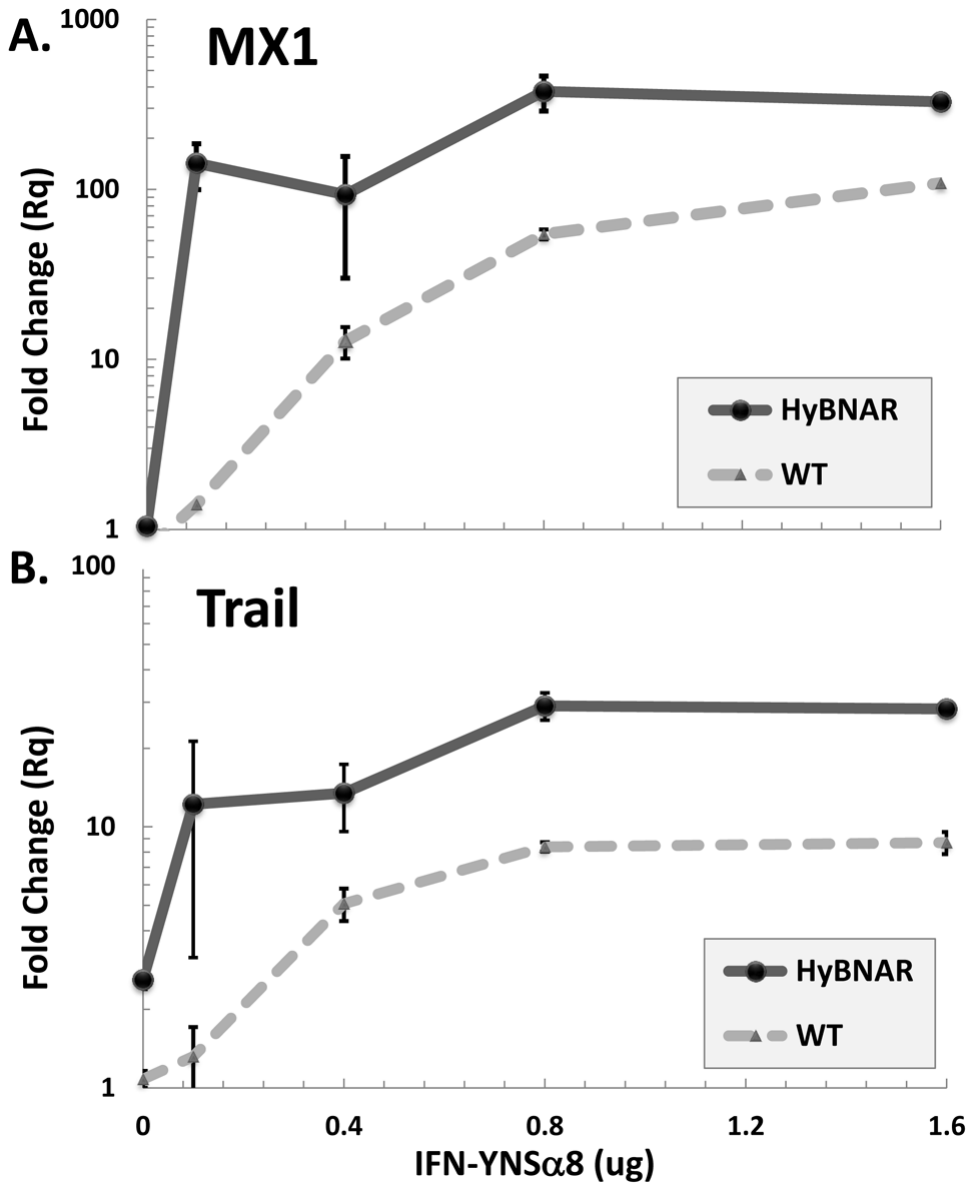
HyBNAR homozygous mice are sensitized to human IFN-I response. Dose response of homozygote HyBNAR after IP injection of increasing doses of the human superagonist IFN-YNSα8. After 6 hours, livers were collected and from the derived RNA/cDNA MX1 (A) and Trail (B) levels were determined by qPCR. Relative fold-change was determined in comparison to untreated wild-type (WT) mice and normalized using the reference gene HPRT1. The average of two mice were used for each injection dosage. The differences in dose response to IFN were significant when comparing control vs. HyBNAR mouse groups (p<0.0005 for both MX1 and Trail expression plots as determined by two-way paired Anova).

The HyBNAR/MX2-LUC used in these studies were doubly heterozygous for both HyBNAR and MX2-LUC. We generated homozygote HyBNAR mice (but without the MX2-LUC transgene) in attempt to further enhance Hu-IFN responsiveness. These homozygous mice were injected with increasing concentrations of IFN-YNSα8, after which livers were tested for gene induction. Wild-type C57BL/6J mice were used as baseline controls. MX1 (which is highly sensitive to IFN-I activation) and TRAIL (which requires higher IFN-I concentrations or higher affinity IFN-Is to be activated) served as probes for IFN-I responsive genes. In the HyBNAR mice, injection of low dose (0.1 ug) of YNSα8 resulted in a 140 and 12-fold increase in MX1 and TRAIL transcripts respectively (Fig. 7). At the highest YNSα8 concentration tested (1.6) µg expression levels of both these genes only increased by another two-fold in relation to 0.1 µg injection. These results demonstrate that the HyBNAR transgenic mouse is sensitive to activation by IFN-I, even at low levels of IFN-I concentrations. Consistent with our previous study, low dose (0.1 ug) of YNSα8 injected into wild-type non-transgenic mice elicited almost no induction in MX1 or TRAIL expression. At the highest dose, of 1.6 µg an induction of 110 and 8.5-fold was measured for these two genes (Fig. 7) in the wild-type mice. From these data we infer at least a 20-fold increased EC_50_ for IFN-I activation in the background of the HyBNAR receptors in comparison to wild-type mice. Thus in summary, these results support that the HyBNAR mice are sensitized to Hu-IFN-I signaling. Sub-optimal activation of the mouse IFNARs is likely taking place as well, especially when higher concentrations of Hu-IFNs are being injected.

### HyBNAR Mice Respond to Human IFN-I in multiple tissues

**Figure 8:**
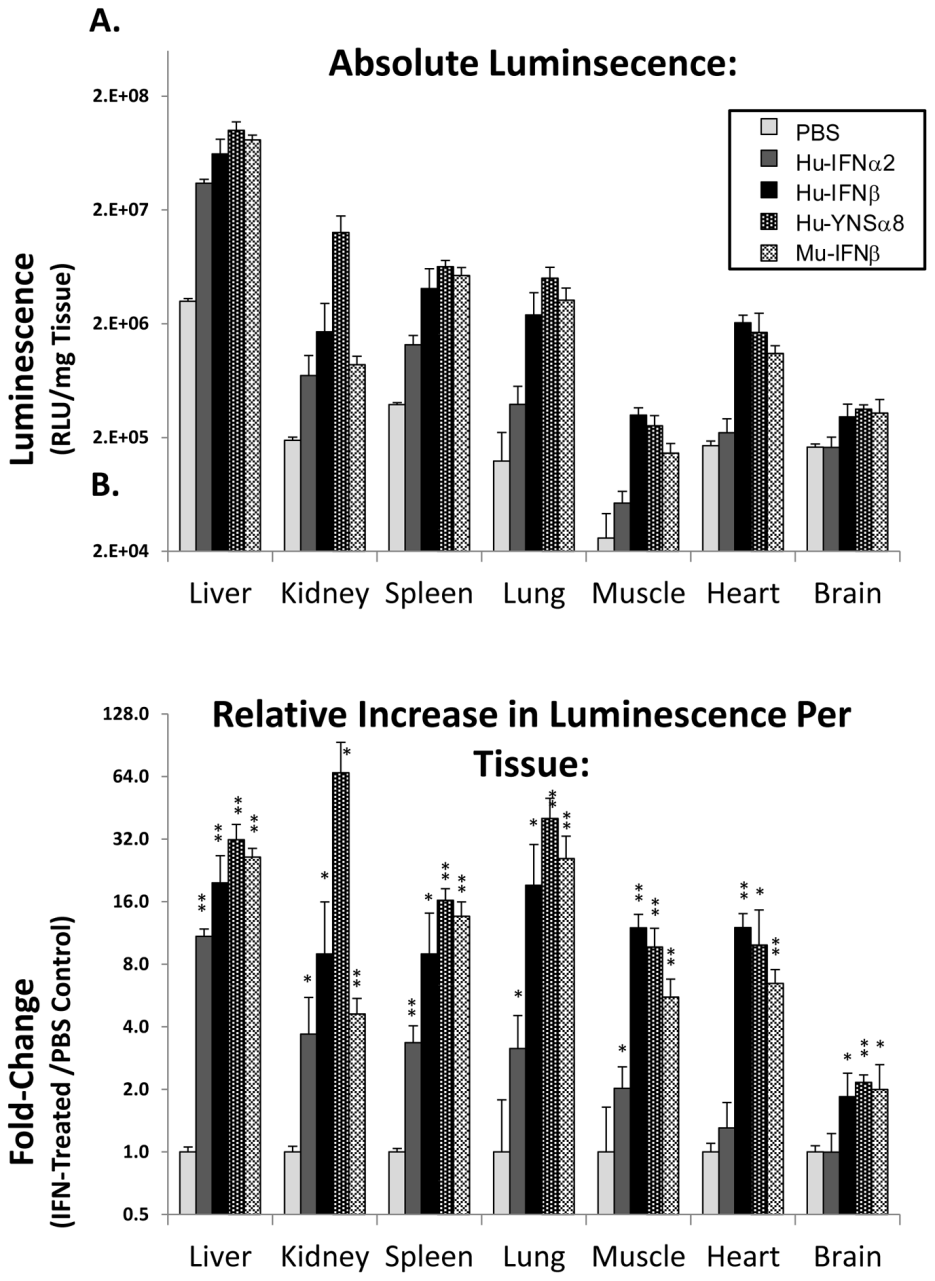
Luciferase activity measurements from tissue homogenates from HyBNAR/MX2-LUC mice. Mice were injected IP with either PBS or 1.0 ug of the indicated type I Interferon. After six hours the mice were perfused with PBS and tissues were collected for homogenization and measured for luciferase activity. (A) Absolute values of luciferase activity standardized per unit wet weight for each tissue. (B) Relative up-regulation of luciferase signal per tissue, in relation to PBS injected controls. Significance values of IFN-treated samples in comparison to PBS controls are shown (one tailed T-Test; *: p≤0.05, **: p≤0.005).

Type I interferon receptors are naturally found on all nucleated cells. To test whether Hu-IFN-I are inducing a widespread response in the HyBNAR mice, we examined their activity by indirect luciferase measurement in a panel of different tissues. Transgenic MX2-LUC/HyBNAR mice were injected IP with 1 ug of either Hu-IFNα2, Hu-IFNβ or YNSα8, along with PBS and Mu-IFNβ as negative and positive controls respectively. Six hours after injections, the mice were perfused with PBS, a selection of tissues were collected, homogenized and then assayed for luciferase activity. Basal MX2-driven luciferase activity measured in control PBS-injected mice (Fig. 8A) was found to be 10–100-fold higher in the liver than in the other tested tissues. This is in agreement with the *in vivo* luciferase imaging data (Figs. 6 & 7) and with previous studies of the MX2-LUC mice [26]. Interferon injections into the HyBNAR/MX2-LUC mice resulted in a 4 to 25-fold increased activity for the different tissues tested, when internally normalized to their PBS-injection controls (Fig. 8B). Brain tissue exhibited the weakest luciferase signal induction with injection of either human or mouse IFN-Is. As the blood-brain barrier is known to restrict the flow of IFN-Is to the central nervous system [27], we assume this to be the cause of the limited activation that we have measured in the brain in response to injected IFNs. When comparing activation of luciferase signal by the human interferons in relation to control Mu-IFNβ, we noted very little difference for all tissues tested, with exception to the kidney, where the activation of the human cytokines was limited. This data therefore support the widespread effects of HyBNAR transgene in different tissues.

### IFN-I induced response in mice is short lived

**Figure 9:**
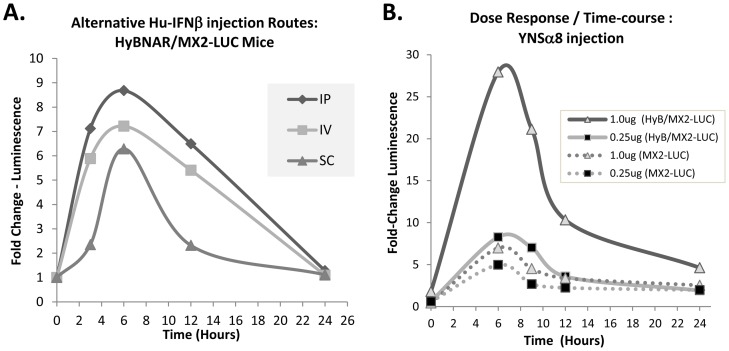
Time and injection regiment affects response to IFN-Is. (A). Response of different injection regiments to 0.2 ug (weight adjusted per 20 g mouse weight) of human IFN**β**. Injections were by intraperitoneal (IP), intra venous (IV) or by sub-cutaneous (SC) route. (B). Mice were injected IP with the indicated increasing doses of YNSα8 and luminosity was measured in a time-course from live animal measurements. MX2-LUC mice doubly transgenic for HyBNAR (solid lines) or without presence of HyBNAR (dotted lines) were both measured. The relative luminosity values given are averaged from three animals (A) or two animals (B) per injection group and were measured from a defined region of maximal signal (found in the liver region) for each mouse as described in Figure S2. In both experiments, baseline luminosity measurements were determined from the mice taken immediately prior to IFN induction.

The serum half-life of IFNβ in humans (pharmacokinetic (PK)) has been reported to range from minutes to a few hours depending on the injection route, with sub-cutaneous injections being the most convenient for patients, and generally showing overall longer half-lives than other routes (reviewed by [28]). Instead of directly measuring the circulating levels of human IFNs in mice, which has been shown to be variable depending on the method of detection used, we used the double transgenic HyBNAR/MX2-LUC mice to follow the pharmacodynamic (PD) activation of the luciferase signal over time. A relatively modest dose of Hu-IFNβ (200 ng/mouse) was injected by intraperitoneal (IP), sub-cutaneous (SC) or intravenous (IV) route and luciferase levels were measured over time. A time-course of semi-quantitative luminescence data was then measured from the live mice (see experimental procedures). We found that in the HyBNAR mice there was no advantage in performing SC injections over the two other injection routes. If at all, the IFN-I induced luciferase signal was delayed and weaker after SC injection (Fig. 9A). These findings concur with other PD studies that we have performed using singly transgenic HyBNAR mice, where IP injections of IFN-Is provided at least as good as, if not better response than the other two injection routes (data not shown). Importantly, irrespective of the route of administration we found that IFNβ-induced MX2-promoter driven luciferase activation returned to baseline within 24 hours of injection (Fig. 9A). This finding in the mice contrasts to PD measurements in humans; a single dose of IFN injection into humans maintained significant levels of MX transcript expression levels even five days post IFNβ injection [29]. To test the importance of dosage and its effects on IFN-I response over time, we injected IP increasing doses of YNSα8 into HyBNAR/MX2-LUC mice and followed luciferase activation by live imaging. High dose of IFN-I (1.0 ug) conferred a strong increase in the amplitude of activation of luciferase signaling, albeit once again, the signal returned to near baseline levels within 24 hours (Fig. 9B). As expected, MX2-LUC mice that do not express the HyBNAR transgene demonstrate minimal IFN-I induced luminescence (Fig. 9B). These data support that in contrast to humans, there is a very limited timeframe of PD response to single injection of IFN-Is in the mouse, and is consistent with published PK studies suggesting a serum half-life of injected IFN-Is in mice of less than one hour [30], [31].

## Discussion

Here we describe for the first time the generation of a transgenic mouse model with the capacity to robustly respond to human IFN-Is. The development of human IFN-Is for clinical use has taken place in spite of the lack of a relevant non-primate preclinical model to adequately assess their function. Despite the discovery of type I IFNs more than 50 years ago and their historically verified clinical use, there remains to this day strong interest for expanded use of IFNs as human therapeutics, as demonstrated by their continued listings as drugs to be tested in new clinical trials (Fig. S2). Currently active clinical trials include not only currently approved IFN drugs for the treatment of new disease indications or in new drug cocktail combinations, but also novel long-life IFN formulations or novel combined drug regiments. Our HyBNAR mice can help the acceleration of such studies, providing a pre-clinical platform for rapid screening of Hu-IFNs before moving to clinical trials. To exemplify this point, we are currently testing a novel long-life variant of the YNSα8 superagonist with promising findings. This study has been greatly facilitated by the availability of the HyBNAR mice in which we can study not only the PD of the superagonist in comparison to IFNβ, but also test its efficacy in a multiple sclerosis disease model environment (*manuscript in preparation*).

**Figure 10:**
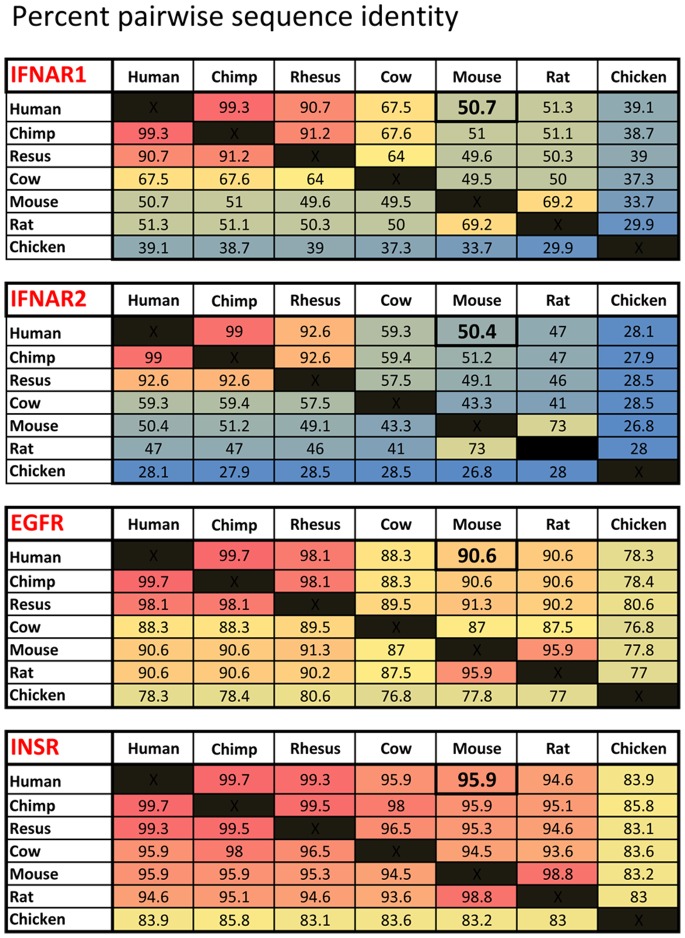
Interspecies Relatedness for a Selection of Cell Surface Receptors. Interspecies protein sequences derived for IFNAR1, IFNAR2, the epidermal growth factor receptor (EGFR) and the Insulin Receptor (INSR) were each compared by respective pairwise BLASTP alignment. Percent amino acid identity scores are given. Pre-calculated percentage identity scores were extracted from the Homologene Server (NCBI, Release #67). Exceptionally, rat IFNAR2 protein sequence (accession #XP_001073550.1) was not available in the Homologene dataset, and was thus curated manually.

For reasons not well understood, interspecies IFNAR sequences have diverged to a much greater extent than that for cognate receptors of other signaling systems, as exemplified by the EGFR or Insulin Receptor signaling pathways (Fig. 10). These interspecies comparisons demonstrate that this divergence is not only constrained to the human-mouse species divide, but extends to other organisms as well. As would be expected, a similar rapid interspecies divergence of sequence extends to the IFN-I cytokines as well [20], [32]. As a result, human IFN-Is are poor activators of IFNAR signaling in non-primate mammals. By the transgenic co-expression of transgenic humanized IFNAR1 and IFNAR2 in the HyBNAR model we can now study the direct effects of Hu-IFN-Is in mice. Using the B16F1-HyBNAR cells, we first demonstrated that the chimeric IFNARs faithfully and sensitively transduce signals after exposure to human IFN-Is. The HyBNAR mice demonstrate strong activation upon Hu-IFN-Is induction across a variety of tested tissues. Hu-IFN-I response was measured by induction of transcription of IFN-response genes as evaluated by direct qPCR (using HyBNAR homozygotes) and by generation of a luciferase signal using a triple-transgenic model, where the HyBNAR mice expressing chimeric IFNAR1 and IFNAR2 were crossed with mice harboring an additional transgene with the MX2 promoter driving luciferase activation (here HyBNAR is heterozygote).

It has been published that the serum half-life of IFN-I in mice ranges from as little as 0.5 hours to as much as 4 hours [30], [31], [33], [34]. The PD data presented in this paper indirectly supports that the PK half-life of IFN-Is in mouse is indeed very short. The resultant effect is that a single-dose injection of IFN-I into the mice provides a pulse of IFN-I activity, but one that is rapidly lost thereafter. This means in real terms, that the pharmacological responses by humans and mice to IFN-I injections are expected to be different, a difference that can be minimized by more frequent injections in mice. Whereas a single dose of IFNβ injection in human induces MX2 activation in blood cells even 120 hours after IFN-I injection [29], in the mouse, we have found that this signal reverts to background levels within 24 hours (Fig. 9). As *in vitro* studies in human cells have shown that some activities of interferon require prolonged time of activation (days of constant IFN exposure [14]), the short half-life in mouse may have significant effects on the outcome of treating disease. In accordance with general considerations of algometric scaling [35], it is not particularly surprising that injected IFN-Is have such a short life-span in mice compared to humans. But the consequence of this difference means that in this respect, the mouse system cannot be simply “humanized”, regardless of presence of the HyBNAR transgene, as single dose injection of non-modified IFN-Is in mice is extremely short lived.

When comparing the levels of gene transcript of the wild-type mouse relative to HyBNAR mouse after activation with either IFN-YNSα8 or Hu-IFNβ we noticed a certain amount of transcription activation also in wild type mouse. This is more evident with increasing concentration of IFN-I injected (Figs. 7 & 8). Whereas in comparing B16F1 to B16F1-HyBNAR cells, the presence of chimeric IFNAR receptors sensitizes the cells to human IFN-Is by three orders of magnitude (Fig. 3), we noted only a 20-fold difference in the EC_50_ of activation of the IFN-response in the HyBNAR mice in comparison to mice without the transgene. Low levels of activation of luciferase signal was also observed in MX2-LUC mice without the HyBNAR transgenes especially after administration of higher levels (1.0 ug) of Hu-IFN-Is. Two possible reasons for the reduced observed species-specificity in animals are: 1) A much higher dose of IFN-Is is typically injected into mice in comparison to humans (typically a 50-fold higher dose in mice after body weight correction [36]), this requirement to inject high doses which may be a consequence to transiently compensate for the rapid clearance of injected IFN-Is in mice. Alternatively, we cannot rule out that endogenous/constitutive Mu-IFNs are priming cells in both wild-type and HyBNAR strains and that even very low activation of the mouse receptor by Hu-IFN leads to a significant effect which is close to saturating levels. This is supported by the fact that at low doses of IFNs the difference is very high (Fig. 7; 0.1 ug values). Despite these potential caveats in our model, it is clear that the presence of the HyBNAR transgene sensitizes mice to Hu-IFN-I signaling, with observed physiological outputs for the transgenic mice expected to emulate the true human effects.

In summary, the generation of the HyBNAR model allows the study of human IFNs in the mouse. This can accelerate *in vivo* study of human IFN-Is as injectable compounds to study added drug effect beyond that of its unaltered endogenous signaling. Moreover, this HyBNAR model can serve as a perfect platform to investigate the relationship between IFNAR receptor levels, IFN-I binding affinity and their potency to the added effects of injected Hu-IFN-Is in cancer and in immune-related diseases. With algometric differences between humans and mice taken into account, the HyBNAR mice provides a convenient non-primate pre-clinical model to advance the study of human IFN-Is.

## Supporting Information

Figure S1 Genomic Genotyping of HyBNAR mouse strains by qPCR. Genomic Tail DNA was subjected to qPCR by TaqMan-like methodology. Assessment of transgenic signal was performed using external probes specific for the HyBNAR transgene and with an internal fluorescent probe. Background signals (IE: amplification from non-transgenic mice) is shown in green. A reference gene encoding mouse TERT was also amplified as to ascertain approximate transgene copy-number for the different transgenic mouse strains.
(TIF)

Figure S2 Cumulative Number of Clinical Trials for Different IFNs. Newly listed clinical trials using IFNα, IFN**β** and (Type II) IFNγ were counted on a year by year basis and cumulative clinical trial numbers over time are plotted. This Data was extracted from ClinicalTrials.gov (a web-based service provided by the U.S. National Institutes of Health). Only the subset of trials listed as “first received” were counted as to avoid possible duplication of same events published over more than one year.
(TIF)
