# Supplementary figures and images for: Bridging the Species Divide: Transgenic Mice Humanized for Type-I Interferon Response
Source: PLoS One. 2014 Jan 9;9(1):e84259. doi: 10.1371/journal.pone.0084259 (PMC3887009; doi:10.1371/journal.pone.0084259)

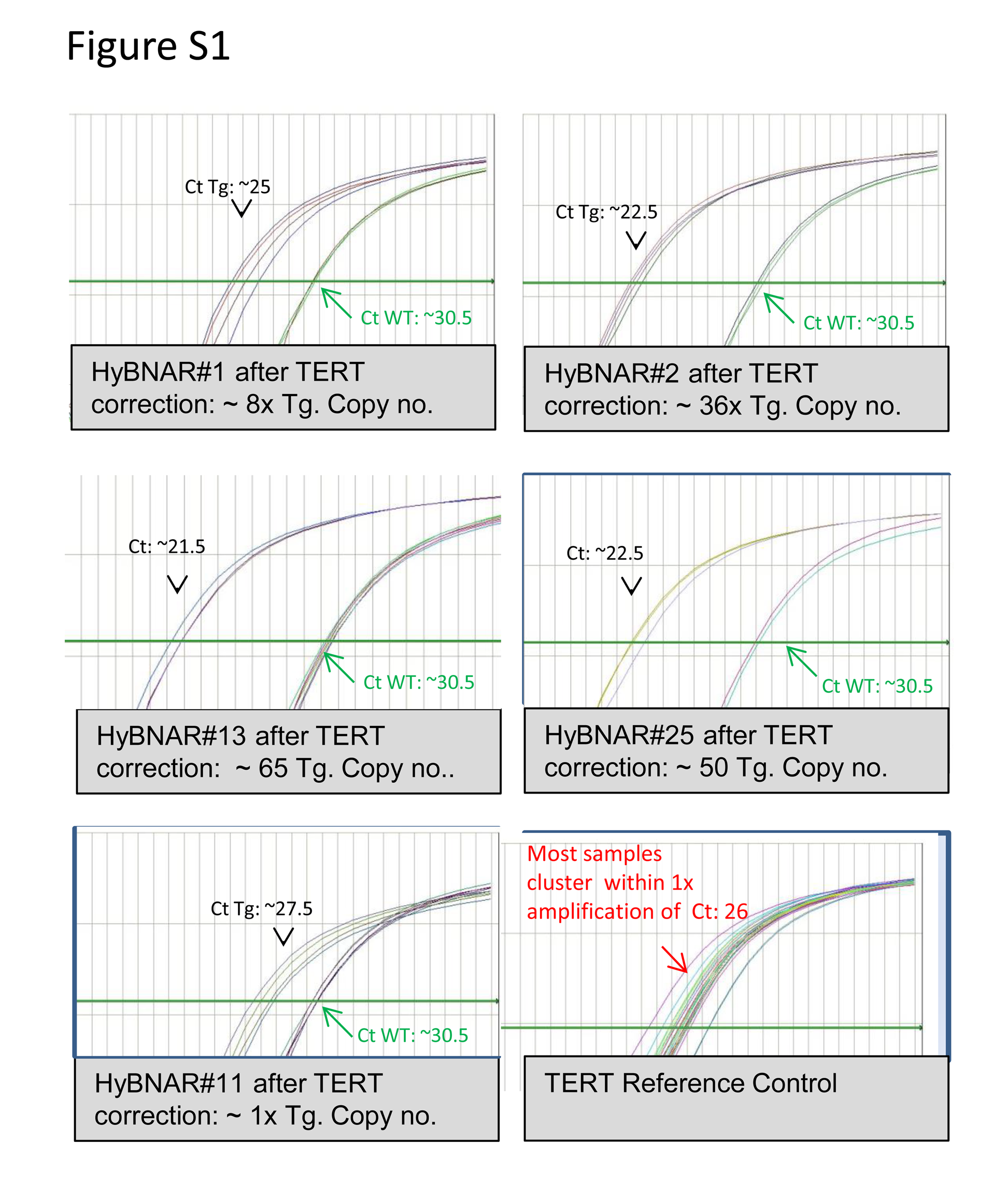

Supplement: Figure S1 — Genomic Genotyping of HyBNAR mouse strains by qPCR. Genomic Tail DNA was subjected to qPCR by TaqMan-like methodology. Assessment of transgenic signal was performed using external probes specific for the HyBNAR transgene and with an internal fluorescent probe. Background signals (IE: amplification from non-transgenic mice) is shown in green. A reference gene encoding mouse TERT was also amplified as to ascertain approximate transgene copy-number for the different transgenic mouse strains. (TIF) [file pone.0084259.s001.tif]

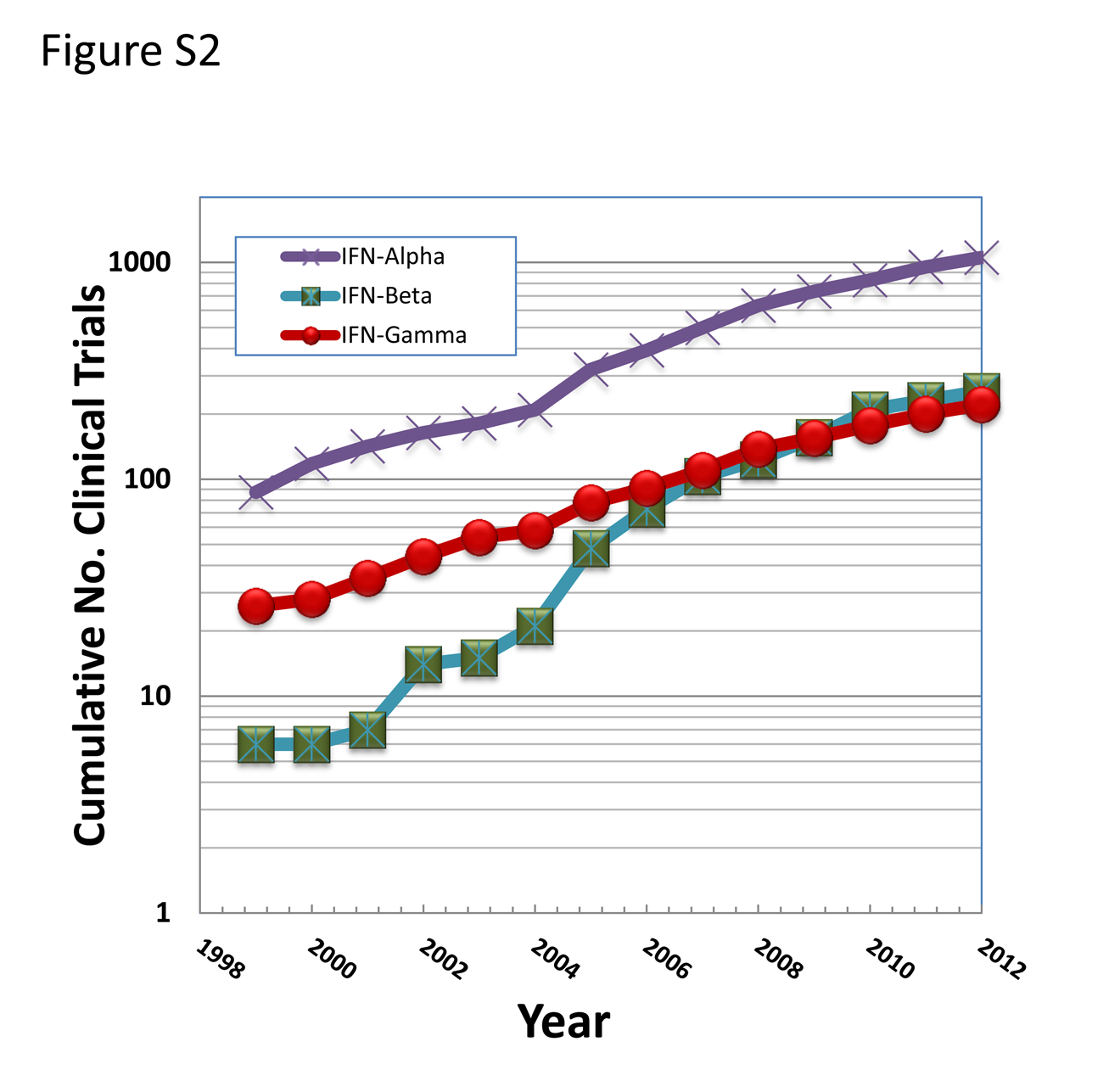

Supplement: Figure S2 — Cumulative Number of Clinical Trials for Different IFNs. Newly listed clinical trials using IFNα, IFNβ and (Type II) IFNγ were counted on a year by year basis and cumulative clinical trial numbers over time are plotted. This Data was extracted from ClinicalTrials.gov (a web-based service provided by the U.S. National Institutes of Health). Only the subset of trials listed as “first received” were counted as to avoid possible duplication of same events published over more than one year. (TIF) [file pone.0084259.s002.tif]
